# Alternate Bearing in Citrus: Changes in the Expression of Flowering Control Genes and in Global Gene Expression in ON- versus OFF-Crop Trees

**DOI:** 10.1371/journal.pone.0046930

**Published:** 2012-10-11

**Authors:** Liron Shalom, Sivan Samuels, Naftali Zur, Lyudmila Shlizerman, Hanita Zemach, Mira Weissberg, Ron Ophir, Eduardo Blumwald, Avi Sadka

**Affiliations:** 1 Department of Fruit Tree Sciences, Agricultural Research Organization, The Volcani Center, Bet Dagan, Israel; 2 Department of Plant Sciences, University of California Davis, Davis, California, United States of America; United States Department of Agriculture, United States of America

## Abstract

Alternate bearing (AB) is the process in fruit trees by which cycles of heavy yield (ON crop) one year are followed by a light yield (OFF crop) the next. Heavy yield usually reduces flowering intensity the following year. Despite its agricultural importance, how the developing crop influences the following year's return bloom and yield is not fully understood. It might be assumed that an ‘AB signal’ is generated in the fruit, or in another organ that senses fruit presence, and moves into the bud to determine its fate—flowering or vegetative growth. The bud then responds to fruit presence by altering regulatory and metabolic pathways. Determining these pathways, and when they are altered, might indicate the nature of this putative AB signal. We studied bud morphology, the expression of flowering control genes, and global gene expression in ON- and OFF-crop buds. In May, shortly after flowering and fruit set, OFF-crop buds were already significantly longer than ON-crop buds. The number of differentially expressed genes was higher in May than at the other tested time points. Processes differentially expressed between ON- and OFF-crop trees included key metabolic and regulatory pathways, such as photosynthesis and secondary metabolism. The expression of genes of trehalose metabolism and flavonoid metabolism was validated by nCounter technology, and the latter was confirmed by metabolomic analysis. Among genes induced in OFF-crop trees was one homologous to *SQUAMOSA PROMOTER BINDING-LIKE* (*SPL*), which controls juvenile-to-adult and annual phase transitions, regulated by *miR156*. The expression pattern of *SPL*-like, *miR156* and other flowering control genes suggested that fruit load affects bud fate, and therefore development and metabolism, a relatively long time before the flowering induction period. Results shed light on some of the metabolic and regulatory processes that are altered in ON and OFF buds.

## Introduction

Alternate bearing (AB) is the process by which cycles of heavy yield (ON crop) one year are followed by a light yield (OFF crop) the next (reviewed in [1]). AB occurs in both deciduous and evergreen fruit and nut tree crops and in forest trees (where it is called “masting”), regardless of their annual reproductive and vegetative cycles. Although in general, the ON and OFF cycles are biennial, in some cases an ON year can be followed by two or more consecutive OFF years, and vice versa. In the classical, most common AB, the OFF year is characterized by low floral intensity (reduced flower number), resulting in low yield, and high vegetative shoot growth, whereas the opposite occurs during the ON year. In some cases, flowering is not limited, but heavy flower and/or fruitlet drop lead to AB. Synchronization among different trees at the plantation/region level is typically initiated by environmental conditions (such as low and high temperatures, water deficit, etc.) that reduce yield. Once initiated, AB becomes entrained through the effect of crop load on endogenous tree factors that ultimately impact the floral intensity; the heavy ON crop reduces return bloom the following spring, whereas the light OFF crop results in an intense return bloom the following spring. Fruit thinning or complete removal (defruiting) as late as September to December of the ON-crop year induces flowering and yield in the following year [2]–[5]. AB has significant economic consequences in many important tree crops. In citrus, during the low-yield OFF year, a significant proportion of the fruit are too large. During the ON year, many small-size fruit with low commercial value are produced.

The mechanism(s) by which the developing crop influences return bloom and yield the following year is not fully understood. Two hypotheses have been suggested. The “nutritional” hypothesis holds that return bloom and yield are proportional to tree carbohydrate status. Lack of carbohydrate in the ON year directly or indirectly reduces flowering the following year [6]. Support for this hypothesis has been provided by showing positive correlations between carbohydrate levels and AB status [7]–[13], whereas others have shown no consistent relationship between tree carbohydrate status and floral intensity at return bloom [2], [3], [14]–[19]. The “hormonal” hypothesis proposes that developing fruit produce an inhibitor that directly or indirectly reduces flowering in the spring following the ON crop [20]–[22]. Although a number of studies have shown correlations between abscisic acid or indole-3-acetic acid and AB status [4], [13], [23]–[26], no direct evidence has been provided for their involvement in the return bloom. Gibberellin (GA) is well-known inhibitor of flowering in citrus; thus, fruit-produced GA has been presumed to be involved in AB [27], [28]. Despite these findings, the roles of carbohydrates and hormones in AB remain unclear and more research is needed to identify factors affecting floral intensity following ON and OFF years. Genetic analysis of AB in apple identified a few QTLs associated with AB, and suggested that hormone-related genes are likely to play a role in the phenomenon [29].

The floral induction period in citrus starts in mid-November and lasts until approximately the end of December to mid-January (Figure S1, the annual cycle in citrus) [30]. Following induction, the bud enters a short resting period, after which the shoot apical meristem differentiates into a floral bud [31], [32]. In parallel to the floral shoot flush, there is a flush of vegetative shoot growth (Figure S2), which continues through June (Spring flush). A second flush of vegetative shoot growth starts in July (Summer flush), and third flush starts in October (Fall flush). Usually, next year flowering occurs mostly on the spring vegetative flush [33]. Flowering in citrus is induced by low temperature, while day length has a relatively minor effect [30]. There is extensive cross-talk between autonomous and vernalization flowering pathways and ample evidence that genes associated with flowering regulation are highly conserved across species [34]. Indeed, citrus genes homologous to *Arabidopsis* flowering control genes most likely possess similar functions. For instance, overexpression of the citrus *FLOWERING LOCUS T* (*FT*), arabidopsis *LEAFY (LFY)* and arabidopsis *APETALA1* (*AP1*) genes in citrus greatly reduced the juvenile period, allowing flowering at the seedling stage [35]–[37]. *FT* was shown to be induced during the annual transition to floral development [38]. In addition, *FT* transcript accumulated in trees subjected to low-temperature floral-inductive conditions [38]. Overexpression of the citrus *LFY*, *AP1* and *SUPPRESSOR OF OVEREXPRESSION OF CONSTANS1* (*SOC1*) genes in *Arabidopsis* resulted in phenotypes similar to those observed when the endogenous genes were overexpressed, and *CsLFY* and *CsAP1* rescued *Arabidopsis* mutants in the respective genes [34], [37]. Similar findings were demonstrated for the citrus *TERMINAL FLOWER* homolog (*CsTFL*) [39]. Inverse relationships were found between fruit load and the expression of *FT, AP1* and *SOC1* in the leaves of ‘Moncada’ mandarin, especially during the flowering induction period [40].

Fruit presence inhibits return flowering. However, it is not clear at which stage the fruit exerts its inhibitory effect: at flowering induction, transition of the shoot apical meristem to floral meristem, or subsequent stages of floral development and bud break. Moreover, the nature of the signal (‘AB signal’) and the organ or tissue from which it originates, be it the fruit itself or the leaf which senses fruit presence, are not known. Regardless of the source tissue for the AB signal, it must be received, directly or indirectly, at the bud, and more specifically, at the apical meristem which has to “decide” whether to develop into an inflorescence or remain a vegetative meristem. Therefore, following perception of the signal, the bud must undergo a series of events which depend on fruit load. In the current work, we analyzed changes in global gene expression during bud development in ON and OFF trees, to identify metabolic and controlling pathways that play a role in bud fate. To determine the earliest time point for the transcriptome analysis, we first analyzed changes in bud morphology during its development, and changes in the expression of key flowering control genes. Based on those results, global gene-expression analysis was carried out at a few key time points in the buds, which receive the ‘AB signal’, and in leaves and stems, which might play a role in generating and transporting the signal.

## Materials and Methods

### Plant material

Plant material was collected from a commercial orchard of 10-year-old Murcott mandarin (*Citrus reticulate* Blanco) trees grafted on sour orange (*Citrus aurantium* L.), located in the central coastal area of Israel, during the years 2009 (an OFF year) and 2010 (an ON year). Although most of the trees in the orchard yielded similarly in a given year, some were exceptional and showed an opposite AB trend. These and nearby trees with the opposite yield status were selected. Overall, nine pairs of trees were chosen, with each three pairs (ON tree and nearby OFF tree) being considered one biological replicate. Comparisons included the two most extremes conditions in regards to chance to flower of buds on the spring flush (Figure S2): fruit-bearing flush of an ON tree and fruitless flush of an OFF tree. About 10 fruitless branches from OFF trees and about 25 fruit-bearing branches from ON trees (Figure S2), collected from the southeast side of the tree, were taken to the laboratory on ice. Leaves and stems and at least 10 buds were removed from the 2 to 3 most distal nodes of one OFF fruitless spring flush (Figure S2) and immediately frozen in liquid nitrogen. Leaves and stems and all buds of a fruit bearing ON spring flush (Figure S2) were removed and immediately frozen in liquid nitrogen. Samples were kept at −80°C until processing. For the genomic analyses, the collections of leaves, stems and buds was as the following in regards to fruit development (Figure S1): mid-May stage I [41], mid- July, early stage II and mid-September, late stage II. For gene expression analyses, samples were collected during the middle of the indicated month. The numbers of inflorescences and vegetative shoots were determined for all of the branches splitting from one major 50- to 60-mm diameter branch located on the southeast side of the tree, during peak blossom, usually the first third of April of the consecutive year. Selection of the sampled branch was performed prior to bud break.

### Light microscopy

Buds were collected and fixed in an FAA solution [10 formaldehyde:5 acetic acid:85 ethanol (70%), v/v]. Fixation was followed by an ethanol dilution series and subsequent stepwise exchange of ethanol with Histoclear (xylem substitute). Samples were embedded in paraffin and cut by microtome (Leica RM2245) into 12-µm sections. Sections were stained with safranin and fast green [42], and examined under a light microscope (Olympus BX50, 50–100× magnification).

### RNA extraction and gene-expression analysis by real-time PCR

Total RNA was extracted from buds and from leaves and stems (LS) using the CTAB extraction method [43]. For buds, approximately 0.2 g of frozen tissue was used, and approximately 2 g of tissue for LS. The volumes of the extraction solutions were adjusted to the amount of starting material. RNA was treated with RQ1 RNase-free DNase (Promega, Fitchburg, WI) according to the manufacturer's instructions. RNA quantity was analyzed in a NanoDrop ND-1000 Spectrophotometer (Wilmington, DE) and RNA quality was determined by Agilent Bioanalyzer (Santa Clara, CA). cDNA was synthesized from 1 µg RNA using OligoT as a primer and M-MLV Reverse transcriptase (Fermentas, Burlington, Ontario, Canada) in a final volume of 25 µl containing the commercially supplied buffer. Primers for the genes *CiFT1/2/3*, *CsAP1, SOC1*, *CsLFY*, *β-actin*, and dual-labeled probes for *CiFT1/2/3* were designed based on genomic and EST sequences (Phytozome, http://www.phytozome.net/, HarvEST, http://harvest.ucr.edu/) using Primer 3 software (Table S1). For the SYBR green reactions, real-time PCR was carried out in a reaction mix containing 2 µM gene-specific forward and reverse primers, 3 µl cDNA (diluted 1∶16), KAPA SYBR FAST qPCR Master Mix (2×) Universal (KAPA Biosystems, Boston, MA), and Ultra-Pure water (Fisher Biotech, Wembley, Australia) in a final volume of 12 µl in a Corbett Rotor-Gene 6000 (Qiagen, Venlo, The Netherlands). Reactions were run for 40 cycles of 10 s at 95°C, 15 s at the annealing temperature for each gene, 20 s extension at 72°C, and the threshold level was determined. For the dual-labeled probe reactions, real-time PCR was carried out in a reaction mix containing 2 µM gene-specific forward and reverse primers, 2.5 µM dual-labeled probes, 3 µl cDNA (diluted 1∶16), TaqMan Universal PCR (2×) Master Mix (Applied Biosystems, Inc., Foster City, CA) and Ultra-Pure water in a final volume of 12 µl in the Rotor-Gene 6000. Reactions were run for 40 cycles of 15 s at 95°C, 60 s annealing and extension at 60°C, and the threshold level was determined. Standard curves were generated for each gene using serial cDNA dilutions. Relative concentration of the product was calculated by the algorithm of the Rotor-Gene software using the CT value. Relative expression (RE) was defined as the ratio between the relative concentration of each gene and that of *β-actin*. The expression of *miR156* was determined using TaqMan® Small RNA Assay Kit (Applied Biosystems) according to manufacturer's instructions; 10 ng total RNA was used, and real-time PCR was run in the Rotor-Gene 6000. The results were normalized against *β-actin*.

### nCounter analysis

The RNA levels of trehalose biosynthetic genes, flavonoid biosynthetic genes, *SQUAMOSE PROMOTER BINDING*-like (*SPL*-like) gene and the reference genes, *β-actin*, *cyclophilin* and *polyubiquitin 2*, were determined by nCounter analysis (Nanostring Technologies, Seattle, WA) at VIB MicroArrays Facility (Leuven, Belgium) according to the manufacturer's instructions [44]. Probe design was based on genomic sequences (http://www.phytozome.net/, Table S2).

### Microarray hybridization analysis

For global gene expression, the citrus GeneChip (Affymetrix, Inc., Santa Clara, CA) carrying 30,171 probes was used. The array is estimated to represent about 15,500 genes. RNA samples were processed as recommended by the Affymetrix GeneChip Expression Analysis Technical Manual at the Center for Genomic Technologies of the Hebrew University of Jerusalem. Total RNA was quantified and then adjusted to a final concentration of 1 µg/µl. Single-stranded and then double-stranded cDNA was synthesized from total RNA (0.5 µg total RNA for each reaction) using oligo-dT primer and the Affymetrix One-Cycle Labeling Kit and control reagents. The resulting double-stranded cDNA was column-purified and then used as a template to generate biotin-tagged cRNA from an *in-vitro* transcription reaction performed with the Affymetrix GeneChip IVT Labeling Kit. The resulting biotin-tagged cRNA (15 µg) was fragmented into strands of 35 to 200 bases in length following published protocols (Affymetrix GeneChip Expression Analysis Technical Manual) and then hybridized at 45°C with rotation for 16 h (Affymetrix GeneChip Hybridization Oven 320) with the Affymetrix Citrus Genome array. The arrays were washed and then stained (EukGE-WS2v5 protocol, p 2.3.11) using SAPE and biotinylated anti-SA in an Affymetrix Fluidics Station 450 followed by scanning in a GeneChip Scanner 3000. Hybridizations were carried out in triplicate, each replicate representing one experimental block. Data processing, including signal analyses, normalization and background subtraction, were carried out using Robust Microchip Analysis (RMA), as described previously [45]. Statistical test for significantly differentially expressed probes was carried out with the Linear Model for Microarray (limmaGUI) as described previously [46].

Gene ontology (GO) analysis was performed using the AgriGo interface (http://bioinfo.cau.edu.cn/agriGO/index.php). Singular enrichment analysis (SEA), which lists enriched GO terms, was used. Differentially expressed probe (DEP) sets were displayed on diagrams of metabolic and other processes using MapMan [47].

### Flavonoid content analysis

Buds (200 to 300 mg) were pulverized in liquid nitrogen using a mortar and pestle and the powder was transferred to a 15-ml tube. Three volumes of water-saturated n-butanol were added, and the mixture was vortexed for a few minutes, then incubated under shaking (200 rpm) for 12 h at room temperature. Following short centrifugation (15,000 RPM at room temperature) and phase separation, the upper phase was collected into a fresh tube, and incubated at room temperature for 1 h to allow the butanol to evaporate. Samples were filtered through a Millex-HV Durapore (PVDF) membrane (0.22 µm) before injection into the LC-MS instrument. MS analyses were carried out by the ultraperformance LC-quadrupole time of flight (UPLC-QTOF) instrument (Waters Premier QTOF, Milford, USA), with the UPLC column connected on-line to a PDA detector (Waters Acquity), and then to the MS detector equipped with an electrospray ion (ESI) source (performed in ESI-positive mode). Separation was performed on a 2.1×50 mm i.d., 1.7-µm UPLC BEH C18 column (Waters Acquity). The chromatographic and MS parameters were as follows: the mobile phase consisted of 0.1% formic acid in water (phase A) and 0.1% formic acid in acetonitrile (phase B). The linear gradient program was: 100% to 95% A over 0.1 min, 95% to 5% A over 9.7 min, held at 5% A for 3.2 min, then returned to the initial conditions (95% A) in 4.2 min. The flow rate was 0.3 ml/min, and the column temperature was kept at 35°C. Masses of the eluted compounds were detected with a QTOF Premier MS instrument. The following settings were applied during the UPLC-MS runs: capillary voltage of 3.2 kV, cone voltage of 30 eV, collision energy of 5 eV, and argon as the collision gas. The following settings were applied during the UPLC-MS/MS run: capillary spray of 3.2 kV, cone voltage of 30 eV, collision energies of 15 to 25 eV, and argon as the collision gas. The m/z range was 70 to 1,000 D. The MS system was calibrated using sodium formate, and Leu-enkephalin was used as the lock mass. MassLynx software version 4.1 (Waters Inc.) was used to control the instrument and calculate accurate masses.

### Statistical analysis

ANOVA test for qPCR results, bud measurements and metabolomic data was conducted using the JMP® version 10 software (SAS Institute Inc. Cary, NC).

## Results

### Flowering intensity and bud size are affected by fruit load

**Figure 1:**
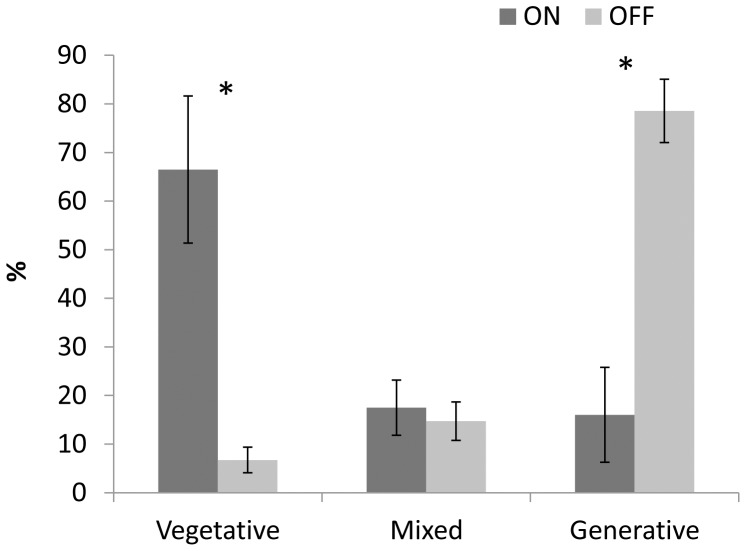
Effect of ON- and OFF-crop years on flowering intensity. Vegetative shoots, generative inflorescences containing only flower buds, and mixed inflorescences containing flowers and leaves, were counted during flowering peak in trees which carried heavy yield (ON) and light yield (OFF) during the previous year. Mean number of three biological replicates ± SE. Stars denote a significant difference between ON and OFF buds (P<0.05).

**Figure 2:**
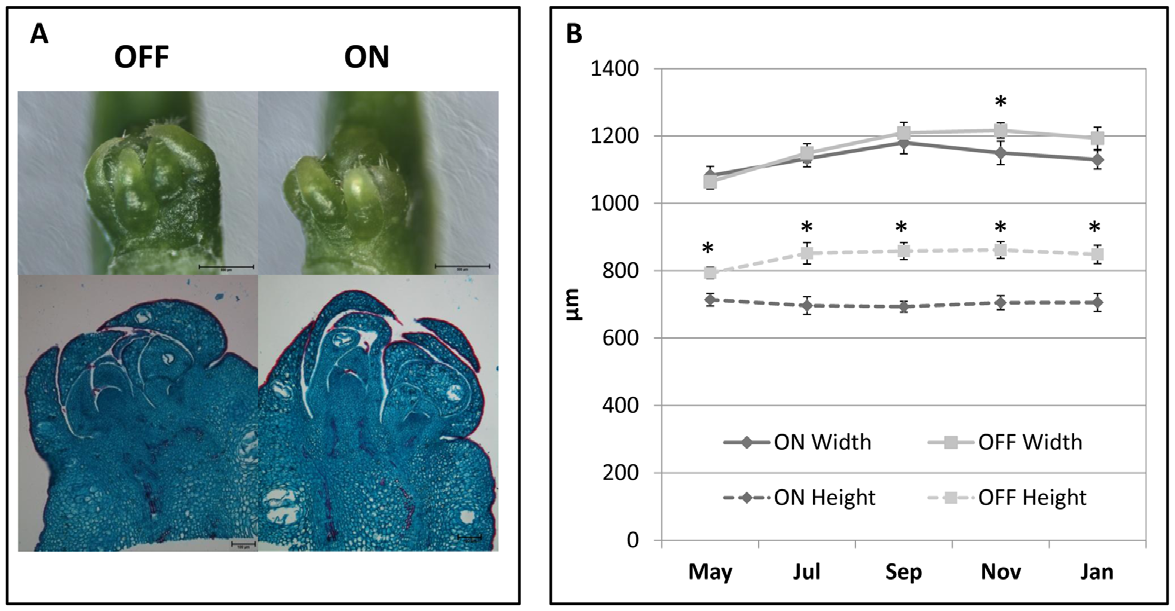
Bud morphology in ON- and OFF-crop trees. Buds were collected from ON- and OFF-crop trees in mid-July (A) and during the indicated months (B), fixed, dissected, dyed and photographed. Bud width and length were measured following photography (B). Mean values of 50 buds ± SE. Stars denote a significant difference between ON and OFF buds during the same time point (P<0.02).

Normally, fruit load status in an AB variety is similar among most of the trees in an orchard in a given year, i.e., most trees either bear a heavy crop (ON-crop year) or a low crop (OFF-crop year). A few trees, however, show the opposite trend, allowing the collection of samples from both AB states from nearby trees. Buds, leaves and stems of heavy-loaded and low-loaded Murcott trees from the same orchard were collected from May, soon after fruit set, until January, the end of the flowering induction period. Flowering intensity of these trees was assessed the following spring (Figure 1). Citrus bears three types of inflorescences: generative (leafless), mixed (leafy, flowers and leaves at various ratios) and vegetative. As expected, in the following spring, ON-crop trees had significantly less generative inflorescences (80% vs. 15%) and more vegetative shoots (65% vs. 5%) than OFF-crop trees. No difference was detected in mixed-type inflorescences. Fruit counting during harvest time showed that ON trees yielded 232±33 fruits/tree, while OFF trees yielded 1542±102 fruits/tree. Buds were measured during the collection period using light microscopy. Usually, there were two adjacent buds in the same position (Figure 2A). External width and height measurements of buds from ON- and OFF-crop trees showed that bud height is slightly induced from May until September, with no difference between ON and OFF-crop buds (Figure 2B). Bud width did not change significantly from May until January, but OFF-crop buds were already significantly larger than ON-crop buds in May. Microscopic analyses of buds from May to January showed no structural differences, with each pair of buds having its own meristem and leaf primordia (not shown).

### Seasonal changes in the expression of flowering genes in buds of ON- and OFF-crop trees

**Figure 3:**
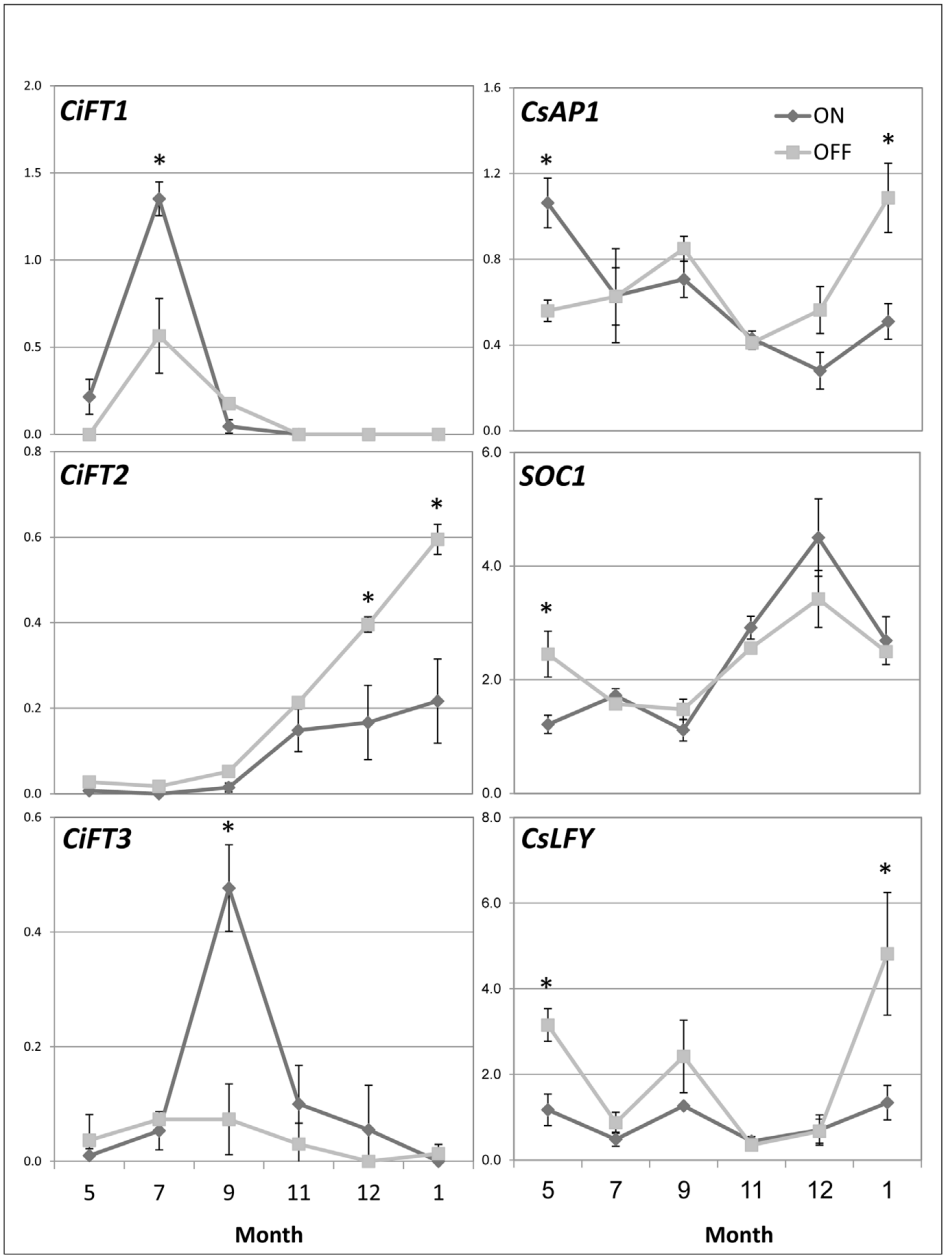
Differences in flowering control genes in ON vs. OFF buds. mRNA levels of the indicated genes in ON and OFF buds were determined by real-time PCR during the indicated months. Mean values of three independent biological replicates ± SE. Stars denote a significant difference between the expression of the gene in ON and OFF buds during the same time point (P<0.05).

The mRNA levels of key flowering genes were measured in buds of ON- and OFF-crop trees at a few time points: mid-May—immediately after fruit set, mid-July—1 month after natural fruit thinning (June drop), and mid-September—the last time point at which fruit removal during an ON-year reverses the AB trend. In addition, samples were collected from mid-November until mid-January, considered the flowering induction period (Figure 3). The following genes were selected (genes names are in accordance with the original work in which they were functionally characterized): *CiFT* [35], *CsAP1, CsLFY* [37] and *SOC1* [34]. Three *CiFT* genes were analyzed. Originally, the expression of three transcripts of *CiFT* were characterized, *CiFT1*, *CiFT2* and *CiFT3*, based on the EST database [38]. However, when comparing the sequences of these three ESTs to the full genome sequence of citrus (http://www.phytozome.org/), it became evident that *CiFT1* and *CiFT2* are most likely encoded by a single gene (*Clementine0.9_023420*), while *CiFT3* is encoded by a different one (*Clementine0.9_033594*) [48]. In addition to these two genes, another gene, highly homologous to *FT*, was found in the genome sequence (*Clementine0.9_023363*) with no representative in the EST database. In the current work, the transcript of *Clementine0.9_023420* is denoted *CiFT1*, that of *Clementine0.9_033594* is denoted *CiFT2* and that of *Clementine0.9_023363* is denoted *CiFT3*.

The mRNA levels of *CiFT1* were significantly induced in ON and OFF buds from May to July, decreased toward September, and remained relatively low during the flowering induction period until January. During May and July, ON buds displayed higher transcript levels than OFF buds. The mRNA levels of *CiFT2* in buds of OFF-crop trees showed a gradual increase of 35-fold overall from September to January. Although gene expression of *CiFT2* in buds of ON trees showed a similar pattern, it was significantly lower than in OFF buds during this period. The expression of *CiFT3* in OFF buds was relatively low and did not change during the tested period. However, in ON buds, it was induced about 10-fold from July to September, and then decreased to levels similar to those of OFF buds from November until January (Figure 3).

In May, the transcript levels of *CsAP1* were about 2-fold higher in buds of ON trees than OFF trees. From July until September, the transcript levels were quite similar in buds from both tree types but, as expected, during the induction period, from November until January, there was a ca. 2.5-fold induction in transcript levels in OFF buds, whereas no such induction was detected in ON buds (Figure 3).

The expression of *SOC1* showed a very similar pattern throughout the tested period in ON and OFF organs. Buds of OFF trees showed a ca. 2-fold increase in mRNA levels relative to ON buds. However, transcript levels were reduced to minimal levels in September, and were then induced about 4-fold in both ON and OFF buds until December, followed by a small reduction toward January (Figure 3).

The expression of *CsLFY* in OFF buds fluctuated during the tested period, with a ca. 4–5-fold increase from December to January. ON buds displayed relatively constant transcript levels during the tested period (Figure 3).

### Changes in global gene expression in ON vs. OFF trees

#### Rationale of the sampling

The above results showed that there was already a clear difference in the sizes of ON and OFF buds in May. Moreover, the mRNA levels of four key flowering control genes,*CiFT1*, *CsAP1*, *SOC1* and *CsLFY*, showed significant differences between ON and OFF buds at that time point. These results thus suggested that changes in metabolic and regulatory pathways between organs of ON and OFF trees can be expected in as early as May, soon after flowering and fruit set. Therefore, global gene-expression analysis was carried out using RNA extracted in mid-May from ON and OFF buds, as the organ which receives the AB signal, and from pooled RNA of leaves and stems (LS), presumably playing a role in generating and transporting the AB signal. We also included an additional time point, mid-September, using RNA from the above organs. Assuming that by the induction period (November through January), the AB signal has already been generated, pooled RNA extracted from buds in December and January was included in the analysis.

#### Hierarchical cluster analysis and statistical analysis of hybridization results

**Figure 4:**
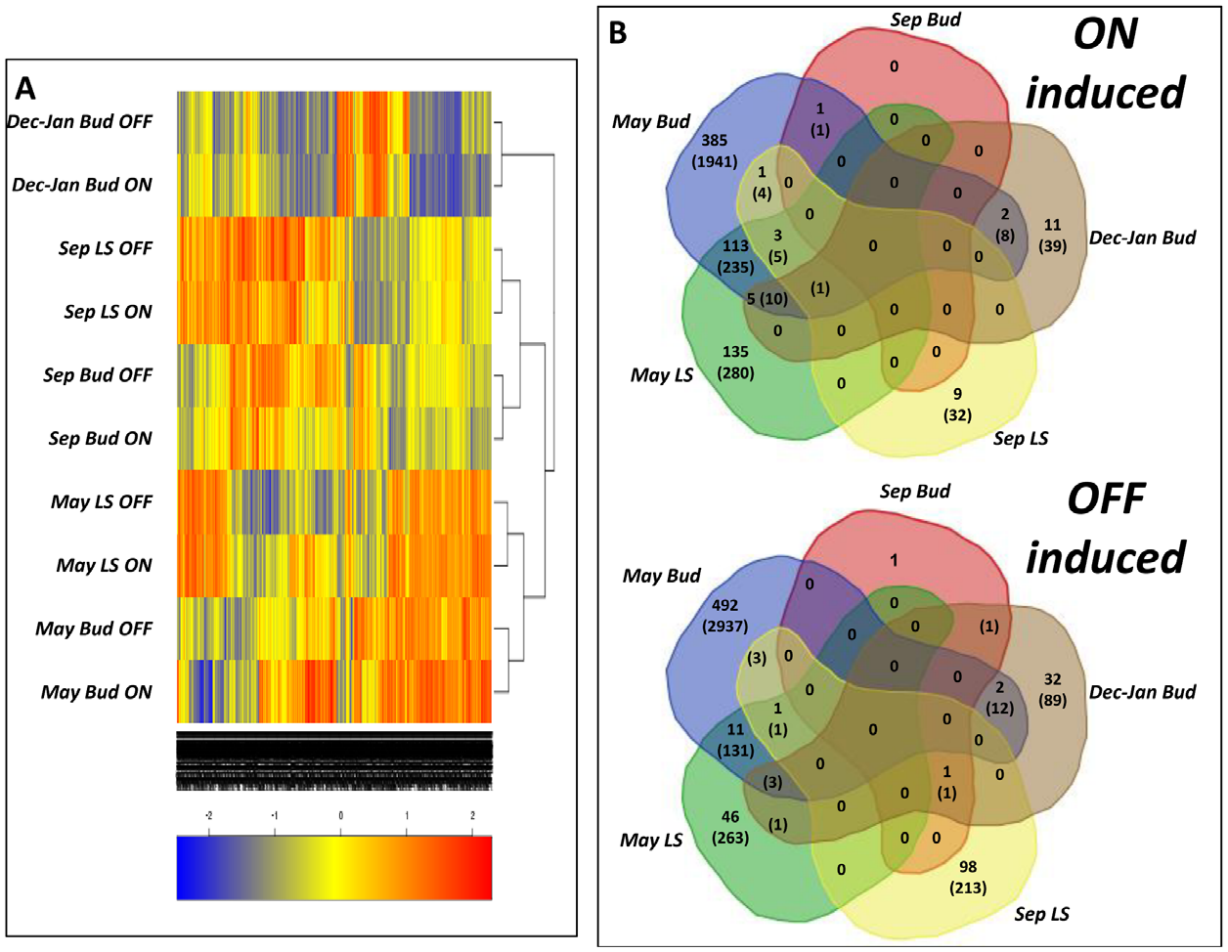
Differences in global gene expression in ON- vs. OFF-crop trees. Hierarchical cluster analysis of global gene expression in buds and leaves+stems (LS) in ON-crop (On) and OFF-crop (Off) trees at the indicated times (A). Venn diagrams of differentially expressed probes, induced in buds and LS of ON- and OFF-crop trees during the indicated months.

Transcriptome analysis was carried out with the above samples using the Citrus Genome Array (Affymetrix) containing 30,171 probes and estimated to represent about 15,500 genes (Table S3). Hierarchical cluster analysis (Figure 4A) showed that the highest level of similarity in the transcription profile was in the same organ between its ON and OFF states at the same time point; different organs at the same time point showed higher levels of similarity in their transcription profiles than the same organs at different time points. Moreover, when comparing the number of DEPs (*P*≤0.05) between buds and LS (regardless of time point and AB state), between ON and OFF states (regardless of time point and organ), and between May and September (regardless of AB state and organ), the highest number was found in the comparison of dates only (15,059); it was lower when comparing tissues only (12,770), and lowest when comparing AB states only (819).

As a comparison of the two AB states was the main target of this study, the number of DEPs in ON and OFF buds and ON and OFF LS at the various time points is presented in Figure 4B. Overall, the highest number of DEPs was detected in May for both buds and LS (6222), while much lower numbers of DEPs were found for buds and LS in September and December–January (263 and 165, respectively). Buds in May displayed the highest number of DEPs: 2205 probes with higher expression in the ON year (of which 510 displayed at least a 2-fold change) and 3087 probes with higher expression in the OFF year (of which 506 displayed at least a 2-fold change). For LS in May, 531 DEPs were found in the ON year (of which 256 displayed at least a 2-fold change) and 399 DEPs in the OFF year (58 displaying at least a 2-fold change). Only 1 and 2 DEPs were found in September buds during ON and OFF years, respectively, whereas the numbers during the induction period were 58 and 107 for ON and OFF years, respectively. In September LS, 42 and 218 DEPs were found in ON and OFF years, respectively. In searching for probes which were significantly (*P*≤0.05) induced in OFF trees, in association with flowering induction, regardless of time or tissue, the probe *Cit.6595.1.s1_at* displayed the highest differential expression. As presented below, this probe was homologous to *SPL* transcription factor from *Arabidopsis*.

### Specific pathways which are altered in ON and OFF trees in May

Considering the high number of DEPs in May relative to the other time points, GO and other analyses were performed only for ON and OFF trees in May. Overall, 1767 (out of 2205) and 2359 (out of 3087) DEPs induced in the ON year and OFF year, respectively, were GO annotated (Table S4). SEA was performed on those probes showing at least a 2-fold change. In addition, all induced and reduced probes in the buds were analyzed by MapMan for altered metabolic and regulatory pathways.

#### Processes induced in OFF trees relative to ON trees

**Figure 5:**
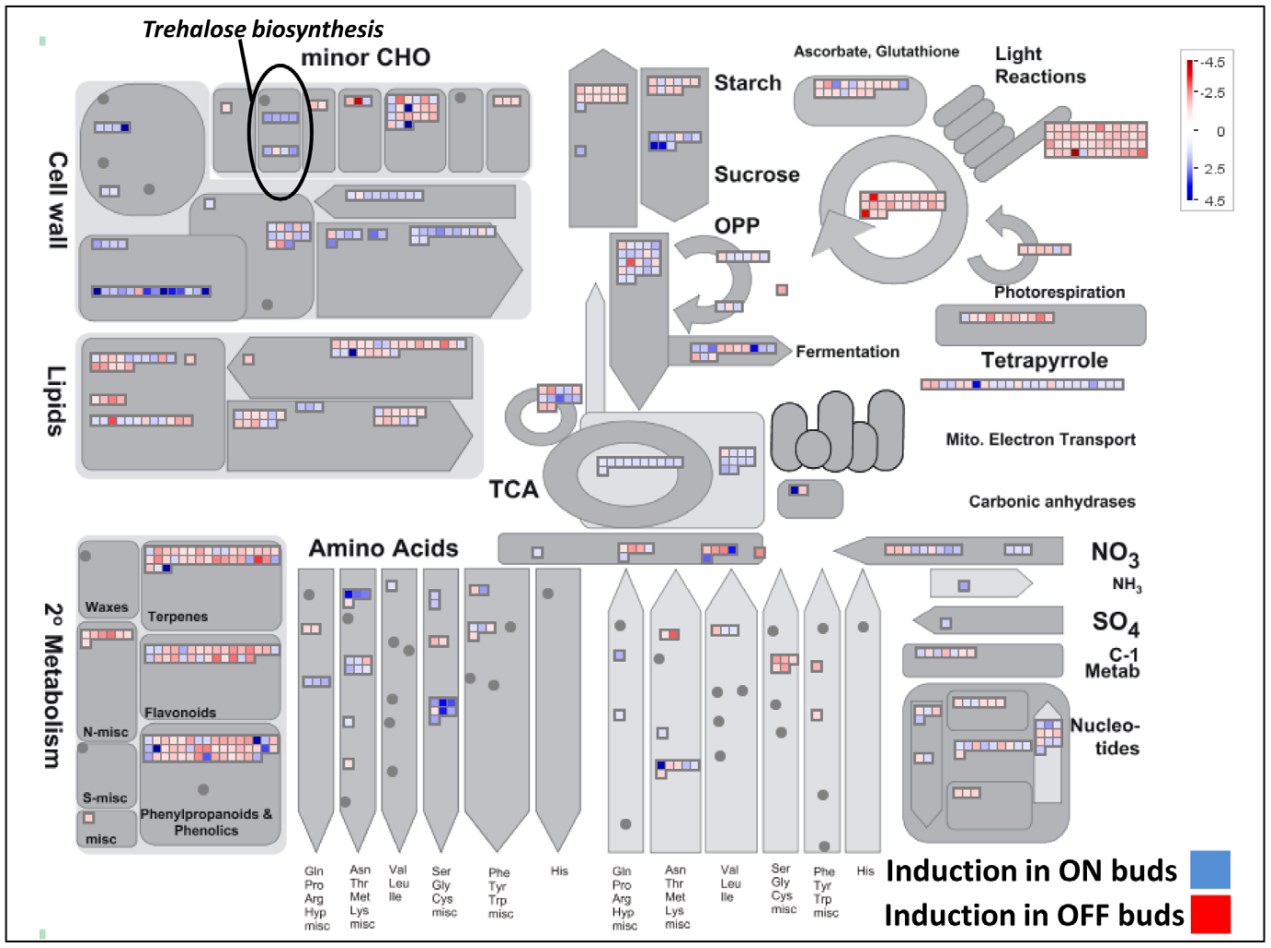
General metabolism in ON and OFF buds. Differentially expressed probes were analyzed using MapMan. Blue squares represent genes induced in ON buds and red squares represent genes induced in OFF buds. A description of the specific genes and their fold change is provided in Table S5.

None of the biological processes which were induced in OFF LS could be identified by SEA—only those in the buds were (Table 1). In buds, major enriched processes included pathways of secondary metabolism, phenylpropanoid, flavonoid and alkaloid metabolisms, and processes related to light and irradiation responses (including red/far red light) and photosynthesis. Analysis of induced and reduced probes by MapMan confirmed these results, as shown in the general metabolism scheme (Figure 5, Table S5). For secondary metabolism, most of the altered probes, belonging to terpene, flavonoid and phenylpropanoid metabolisms, were induced in the OFF buds. Metabolic pathways for some amino acids, such as serine, glycine, and cysteine, were also induced in the OFF buds. Strikingly, most of the probes belonging to the photosynthetic pathways, light reactions, photorespiration and Calvin cycle were significantly induced as well (Figure S3, Table S6). For starch metabolism, the picture seemed more complex, as both probes belonging to starch catabolism and synthesis were induced (Figure 5). Probes for the TCA cycle were moderately induced.

**Table 1:** Gene ontology (GO) categorization of genes induced in OFF buds.

GO term	Description	% in input list	% in BG/Ref	p-value	FDR	Fold enrichment
GO:0019748	secondary metabolic process	5.5336	1.2429	7.00E-10	6.60E-07	4.45
GO:0009698	phenylpropanoid metabolic process	3.1621	0.5005	2.20E-08	6.70E-06	6.32
GO:0009812	flavonoid metabolic process	2.5692	0.3149	2.00E-08	6.70E-06	8.16
GO:0009699	phenylpropanoid biosynthetic process	2.5692	0.4707	2.20E-06	0.00052	5.46
GO:0009813	flavonoid biosynthetic process	1.9763	0.3016	6.30E-06	0.0011	6.55
GO:0010017	red or far-red light signaling pathway	1.1858	0.0895	7.30E-06	0.0011	13.25
GO:0009639	response to red or far-red light	1.7787	0.2519	9.70E-06	0.0013	7.06
GO:0009821	alkaloid biosynthetic process	1.1858	0.1061	2.10E-05	0.0024	11.18
GO:0009820	alkaloid metabolic process	1.9763	0.3546	2.60E-05	0.0027	5.57
GO:0019438	aromatic compound biosynthetic process	2.9644	0.8120	5.10E-05	0.0048	3.65
GO:0009585	red, far-red light phototransduction	0.9881	0.0795	6.00E-05	0.0051	12.42
GO:0009583	detection of light stimulus	0.9881	0.0862	9.00E-05	0.0065	11.47
GO:0007602	phototransduction	0.9881	0.0862	9.00E-05	0.0065	11.47
GO:0006725	cellular aromatic compound metabolic process	4.5455	1.7699	0.00017	0.011	2.57
GO:0009582	detection of abiotic stimulus	0.9881	0.0994	0.00018	0.011	9.94
GO:0009581	detection of external stimulus	0.9881	0.1027	0.00022	0.013	9.62
GO:0051716	cellular response to stimulus	3.3597	1.1435	0.00024	0.013	2.94
GO:0042398	cellular amino acid derivative biosynthetic process	2.7668	0.8584	0.00032	0.017	3.22
GO:0009809	lignin biosynthetic process	1.1858	0.1823	0.00046	0.022	6.50
GO:0009808	lignin metabolic process	1.1858	0.1823	0.00046	0.022	6.50
GO:0006575	cellular amino acid derivative metabolic process	3.3597	1.2495	0.00064	0.028	2.69
GO:0009628	response to abiotic stimulus	4.7431	2.0815	0.00067	0.028	2.28
GO:0009791	post-embryonic development	2.5692	0.8352	0.00078	0.03	3.08
GO:0009416	response to light stimulus	2.5692	0.8319	0.00075	0.03	3.09
GO:0051606	detection of stimulus	0.9881	0.1359	0.00082	0.031	7.27
GO:0015979	photosynthesis	1.7787	0.4574	0.00096	0.034	3.89
GO:0009314	response to radiation	2.5692	0.8651	0.0011	0.037	2.97
GO:0009805	coumarin biosynthetic process	0.9881	0.1525	0.0014	0.045	6.48
GO:0009804	coumarin metabolic process	0.9881	0.1525	0.0014	0.045	6.48

BG, background; Ref, reference; FDR, false discovery rate.

#### Processes induced in ON trees relative to OFF trees

Only one process was induced in LS during the ON year—Cell Wall Organization (GO:0009664)—which showed about 18-fold enrichment (*P* = 2.4E-7; false discovery rate = 0.0012). Similarly, in ON buds, the metabolism of glucan, a cell-wall component, showed induction (Table 2, Figure 5, Table S5). Other processes induced in the ON buds were involved in disaccharide and polysaccharide metabolism (Table 2), including trehalose and sucrose metabolisms (Figure 5, Table S5).

**Table 2:** Gene ontology (GO) categorization of genes induced in ON buds.

GO term	Description	% in input list	% in BG/Ref	p-value	FDR	Fold enrichment
GO:0010252	auxin homeostasis	0.9804	0.0331	6.10E-07	0.0005	29.58
GO:0006073	cellular glucan metabolic process	3.1373	0.7292	7.80E-06	0.0011	4.30
GO:0009312	oligosaccharide biosynthetic process	1.1765	0.0829	6.50E-06	0.0011	14.20
GO:0044042	glucan metabolic process	3.1373	0.7292	7.80E-06	0.0011	4.30
GO:0005992	trehalose biosynthetic process	0.9804	0.0464	4.50E-06	0.0011	21.13
GO:0046351	disaccharide biosynthetic process	1.1765	0.0762	3.80E-06	0.0011	15.43
GO:0044264	cellular polysaccharide metabolic process	3.1373	0.7524	1.20E-05	0.0014	4.17
GO:0005976	polysaccharide metabolic process	3.5294	0.9380	1.40E-05	0.0014	3.76
GO:0005991	trehalose metabolic process	0.9804	0.0597	1.80E-05	0.0017	16.43
GO:0005984	disaccharide metabolic process	2.5490	0.5966	5.60E-05	0.0046	4.27
GO:0009311	oligosaccharide metabolic process	2.5490	0.6099	7.00E-05	0.0053	4.18
GO:0042221	response to chemical stimulus	6.8627	2.9598	0.00011	0.0079	2.32
GO:0016137	glycoside metabolic process	2.5490	0.6529	0.00014	0.0088	3.90
GO:0005985	sucrose metabolic process	2.3529	0.5734	0.00015	0.0091	4.10
GO:0005982	starch metabolic process	2.3529	0.6032	0.00025	0.014	3.90
GO:0044262	cellular carbohydrate metabolic process	4.3137	1.6075	0.00027	0.014	2.68
GO:0016138	glycoside biosynthetic process	1.1765	0.1624	0.00035	0.017	7.24
GO:0010035	response to inorganic substance	1.5686	0.3281	0.00068	0.031	4.78
GO:0010038	response to metal ion	1.3725	0.2585	0.00078	0.034	5.31
GO:0009733	response to auxin stimulus	1.9608	0.5204	0.001	0.042	3.77

BG, background; Ref, reference; FDR, false discovery rate.

### Expression of citrus *SPL*-like and *miR156*

**Figure 6:**
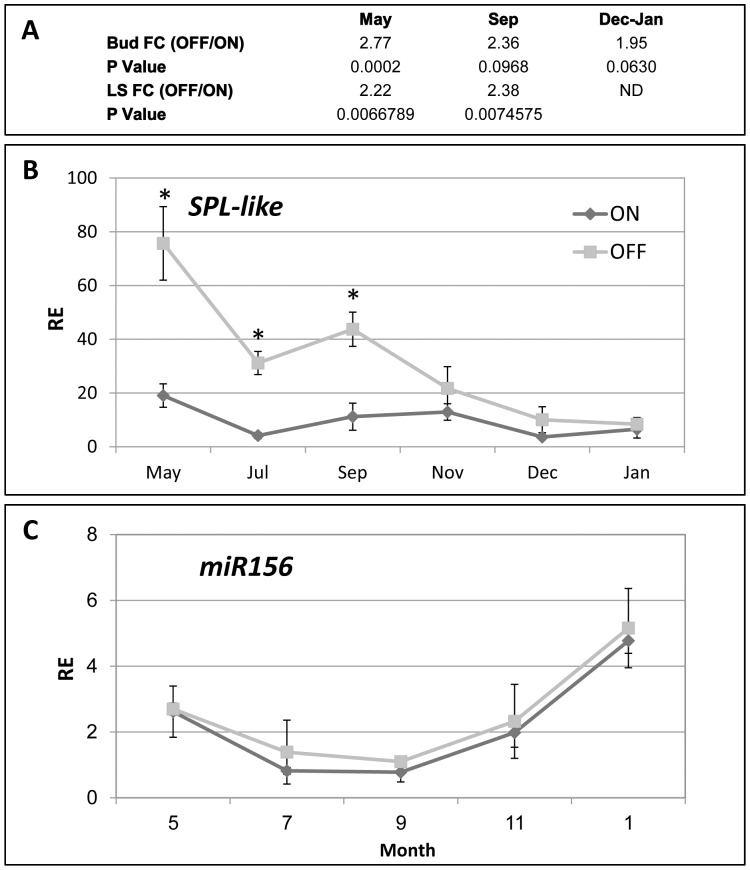
Expression of *SPL*-like and *miR156* in ON and OFF buds. Fold change (FC) between OFF and ON buds and leaves+stems (LS) of microarray probe Cit corresponding to *SPL*-like (A) in the indicated months. mRNA of *SPL*-like (B) and *miR156* (C) was analyzed in ON and OFF buds during the indicated months. Mean number of three biological replicates ± SE. Stars denote a significant difference between the expression of the gene in ON and OFF buds during the same time point (P<0.05).

As already mentioned, an *SPL*-like probe showed the highest induction level in OFF vs. ON LS and buds at all tested time points. *SPL* genes make up a family of transcription factors which have been previously shown to affect flowering time and phase change in *Arabidopsis* [49]. The citrus *SPL*-like gene showed the highest homology to *SPL3, SPL4* and *SPL5* from *Arabidopsis*. Members of the *Arabidopsis SPL* gene family contain a *miR156*-binding site, and direct evidence has been provided that *miR156* represses the expression of some of them [50]. A putative *miR156*-binding site was present in the 3′UTR of the citrus *SPL*-like gene. Expression of the citrus *SPL*-like gene was analyzed in ON and OFF buds throughout the year using nCounter technology (Figure 6). In OFF buds, its expression was reduced from May until the induction period. As expected, the expression in OFF buds was significantly higher than in ON buds from May to December. The expression pattern of *miR156* was also investigated in ON and OFF buds. No difference was detected between them, but the expression was slightly reduced from May until September and then induced from September and throughout the flowering induction period, in correlation with the repression in *SPL*-like gene expression.

### Expression analyses of genes of trehalose and flavonoid metabolisms, and metabolomic analyses of flavonoids

**Figure 7:**
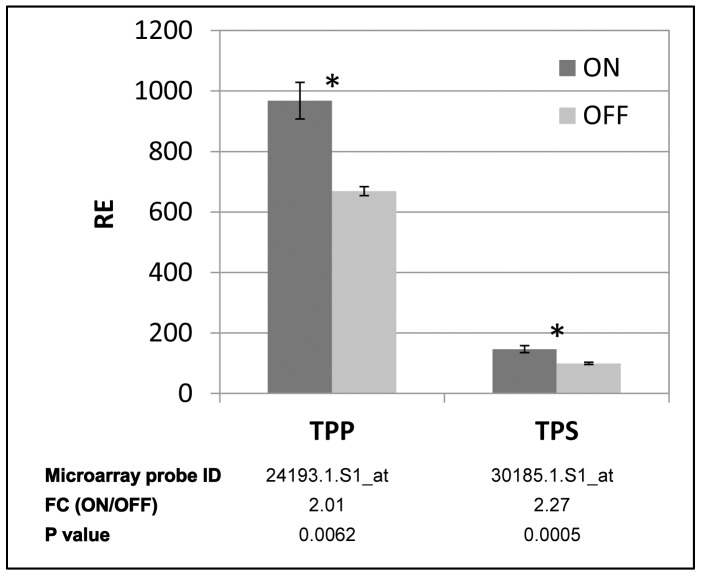
Expression of trehalose metabolism genes in ON and OFF buds. mRNA levels of trehalose phosphate phosphatase (TPP) and trehalose phosphate synthase (TPS) were measured in ON and OFF buds in May. Fold change (FC) between ON and OFF buds in their corresponding microarray probes are shown in the lower panel. Mean number of three biological replicates ± SE. Stars denote a significant difference in the expression of the gene between ON and OFF buds (P<0.05).

Global gene-expression analysis showed that probes encoding trehalose metabolism enzymes are induced in ON buds (Figure 7, lower panel). Validation of the microarray results by nCounter technology revealed that the two genes of trehalose metabolism, encoding trehalose phosphate phosphatase (*TPP*) and trehalose phosphate synthase (*TPS*), are indeed induced in ON buds in May, although the fold-change was lower than that detected for their corresponding microarray probes (Figure 7, upper panel). During the following months, no significant change in these two genes' expression was detected between ON and OFF buds, and their pattern of expression was different, especially from September to January (Figure S4).

**Figure 8:**
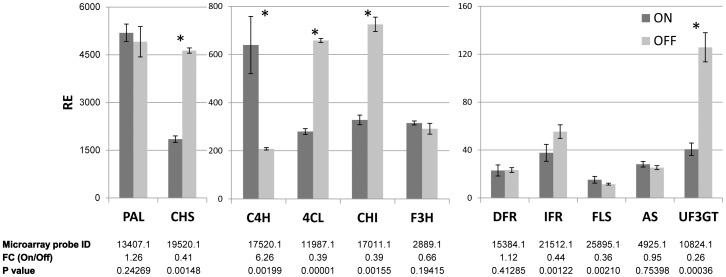
Expression of flavonoid biosynthetic pathway genes in ON and OFF buds. The mRNA levels of phenylalanine ammonia-lyase (PAL), chalcone synthase (CHS), cinnamate 4-hydroxylase (*C4H*), 4-coumarate:coenzyme A ligase (*4CL*), chalcone isomerase (CHI), flavanone 3-hydroxylase (*F3H*), dihydroflavonol 4-reductase (DFR), isoflavone reductase (*IFR*), flavonol synthase (FLS), Anthocyanidin synthase (*AS*) and UDP-glucose:flavonoid-3-O-glucosyltransferase (*UF3GT*) were measured in ON and OFF buds in May. Fold change (FC) between ON and OFF buds in their corresponding microarray probes are shown in the lower panel. Mean number of three biological replicates ± SE. Stars denote a significant difference in the expression of the gene between ON and OFF buds (P<0.05).

Probes for six genes of the flavonoid metabolic pathway, 4-coumarate:coenzyme A ligase (*4CL*), chalcone synthase (*CHS*), chalcone isomerase (*CHI*), isoflavone reductase (*IFR*) flavonol synthase (*FLS*), and UDP-glucose:flavonoid-3-O-glucosyltransferase (*UF3GT*) were induced in OFF buds in May, whereas the probe for one gene, cinnamate 4-hydroxylase (*C4H*), was reduced in these buds (Figure 8, lower panel). Validations were therefore carried out for the 11 genes of the flavonoid biosynthetic pathway (Figure 8, upper panel). As expected, genes encoding 4CL, CHS, CHI and UF3GT showed significantly higher expression (more than 2-fold) in buds of OFF trees as compared to those of ON trees. One gene, *IFR*, showed only marginal induction in OFF buds, as compared to 2-fold induction in the microarray results. The analysis also confirmed the transcriptome analysis result that the gene for C4H is induced in ON buds. However, as opposed to the microarray results, one gene, *FLS*, showed no significant induction in the nCounter analysis. Other genes of the flavonoid biosynthesis pathway, phenylalanine ammonia-lyase (*PAL*), dihydroflavonol 4-reductase (*DFR*), flavanone 3-hydroxylase (*F3H*) and Anthocyanidin synthase (*AS*), showed no change in their transcript levels between ON and OFF buds. The expressions of all 11 genes were analyzed from May until January, but most of them showed no significant change between ON and OFF buds at the rest of the time points (Figure S5).

**Figure 9:**
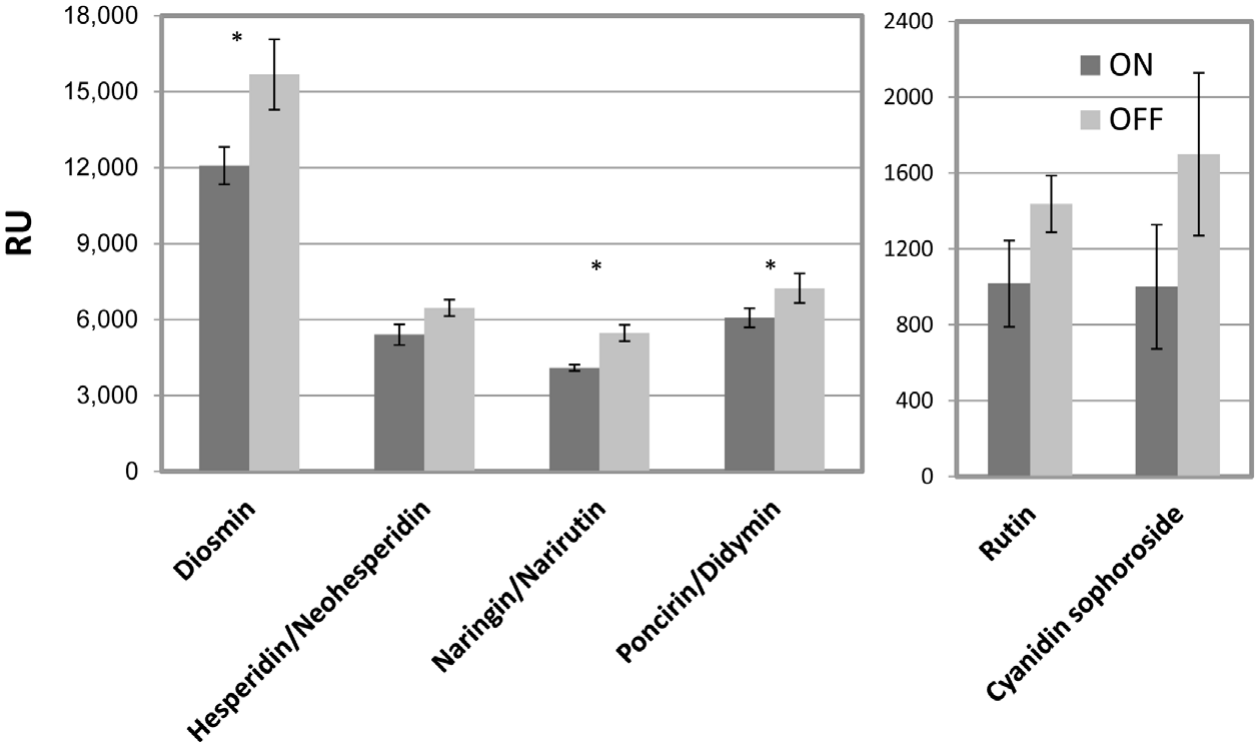
Flavonoid content in ON and OFF buds. The indicated flavonoids were measured in ON and OFF buds using LC-MS. Y axis indicates the intensity of each compound, RU relative units. Mean number of three biological replicates ± SE. Stars denote a significant difference between ON and OFF buds (P<0.05).

The flavonoid biosynthetic pathway was further investigated by metabolomic analysis of a few flavonoids in ON and OFF buds during May using UPLC-QTOF-MS. The following compounds were identified by accurate mass, fragmentation pattern and a few standards: naringin/narirutin, hesperidin/neohesperidin, poncirin/didymin (flavonones), diosmin (flavone). In agreement with the gene-expression analyses, the intensities of all tested compounds were higher in OFF buds than ON buds, although with varied significance (Figure 9), suggesting that flavonoid biosynthesis is induced in OFF buds, allowing an increase in these four flavonoid groups.

## Discussion

### Bud population and morphology in ON and OFF trees

The effect of year 1 yield on the return bloom of year 2 was as expected: heavy yield resulted in a lower number of flowers and higher number of vegetative buds, whereas the opposite was true following a light yield (Figure 1). Overall, buds collected during year 1 from OFF trees had a ca. 95% chance of flowering, as compared to buds collected from ON trees which had a ca. 30% chance of flowering. No effect of year 1 yield was detected on mixed-type shoots (inflorescences containing flowers and leaves at various ratios), only on generative buds (inflorescences carrying only flowers with no leaves). Indeed, while fruit load and flowering manipulations by various means, such as defruiting, GA treatment and fall girdling, are highly effective on generative inflorescences, their effect on mixed-type shoots is not always significant [3], [5], [15], [16], [51]–[53].

In general, bud morphology and anatomy did not change significantly from May to January. This is in agreement with Lord and Eckerd's [31] original finding of microscopic bud break only being detectable in as late as the end of December, and macroscopic bud break being detectable about 2 weeks after that. However, in May, soon after flowering and fruit set, OFF buds were already significantly longer than ON buds. In Pixie mandarin, fruit has been shown to inhibit vegetative shoot development by reducing both their number and the number of nodes which can bear floral and vegetative shoots the following spring [5]. A reduction in the number of nodes during the ON year might well explain the difference in bud length between ON and OFF trees.

### Expression of flowering control genes in ON and OFF buds

To date, the expression of flowering control genes has been mostly investigated in leaves and stems. To the best of our knowledge, this work provides the only report describing the expression of flowering control genes in citrus buds. We recently demonstrated that during the flowering induction period, the mRNA levels of *FT* were considerably higher in buds than in leaves (Goldberg-Moeller R, Shalom L, Shlizerman L, Samuels S, Zur N, Ophir R, Blumwald E, Sadka A, Submitted). The mRNA levels of *FT2*, as well as of *AP1* and *LFY*, were higher in OFF buds than in ON buds during the flowering induction period, similar to that which has been described for *FT* and *AP1* in Moncada mandarin ON and OFF leaves [40]. Similarly, the expression of *FT*, *AP1* and *LFY* in mango was induced in leaves during the flowering induction period, with OFF trees showing higher levels of expression of *FT* and *AP1* [54]. Therefore, these results suggest that *FT2*, *LFY* and *AP1* might be involved in the annual phase transition in citrus. As the number of studied cases is so far too small, it is still difficult to generalize this picture to other perennial fruit trees [55]. Moreover, in apple, a deciduous tree, the expression of *AP1* and two *FT* genes was usually higher on fruit-bearing shoots than in succulent shoots [56]. The expression patterns of *CiFT1* and *CiFT3* provide similar picture to the two apple genes and a different one to *CiFT2*: first, ON buds displayed higher levels of their transcripts, and second, their induction did not occur during the flowering induction period, as with the other genes. In poplar, one of the *FT* paralogs' involvement in vegetative growth has recently been demonstrated [57]. It is therefore possible that *CiFT1* and *CiFT3* are involved in the control of vegetative rather than reproductive growth. There are three vegetative flushes in citrus: spring flush (February–March), summer flush (June–July) and fall flush (October–November). However, ON trees display suppressed vegetative growth. Therefore, we suggest that *CiFT1* and *CiFT3* either play a role in the suppression of vegetative flush development, or help determine ON bud fate toward vegetative growth the following spring. If the latter is indeed the case, the early induction of *CiFT1* and *CiFT3* should generate a signal that persists for a long time. If such a signal is indeed generated, then it should also be considered to be reversible, as the defruiting of ON trees induces flowering the following spring. Obviously, expression patterns provide only coincidental evidence for the involvement of the above genes in phase transitions. More direct evidence, such as that provided to establish the involvement of *FT*, *LFY* and *AP1* in the juvenile-to-adult phase transition, is required. However, one reasonable scenario (among others) is that *CiFT2*, *AP1* and *LFY* are induced in OFF buds and leaves in response to flowering-permissive environmental and endogenous signals. In ON organs, high fruit load prevents or reduces their induction by generating a ‘negative AB signal’. The nature of the endogenous signal affected by fruit load, be it nutritional status of the tree, hormonal or some other signal(s), is currently unknown. However, while considering the various possibilities, the following points should be borne in mind: (1) like in *Arabidopsis*, it is reasonable to assume that more than one flowering control pathway exists in citrus [58], [59], and therefore the effect of fruit load might be exerted on more than one pathway; (2) fruit load might also act via some exogenous signal, such as low temperature. As already noted, flowering in citrus is induced by low temperature, while day length has a relatively minor effect [30]; under permissive temperatures, shortening day length might induce more flowers, but has no effect under non-permissive temperatures. In *Arabidopsis*, the vernalization-flowering promotion pathway is dependent on the removal of *FLOWERING LOCUS C* (*FLC*) inhibition of *FT* expression in the leaves, and on the expression of *FD* and *SOC1* in the apical meristem [60]. However, to the best of our knowledge, *FLC*-like genes have not yet been described in plants other than crucifers, questioning the validity of the *Arabidopsis* mechanism in fruit trees. Moreover, although *SOC1* was induced during the flowering induction period (at least from September to December), no difference was detected between ON and OFF buds, in contrast to the case in leaves; (3) although day length has only a minor effect on flowering induction in citrus, day-length shortening, rather than temperature drop, might explain the 6-fold induction in *FT* expression from May to September in the OFF buds; (4) expression patterns are not always easily interpreted. For example, *AP1* expression was higher in ON than OFF buds in May, whereas that of *LFY* and *SOC1* was higher in OFF than ON buds. It might be that these genes have other functions at this stage.

One of the outcomes of the genomic analysis, validated by real-time PCR, was the induction in OFF buds and LS of the *SPL*-like gene (Figure 6). SPLs play a role in the juvenile-to-adult and annual phase transitions and are regulated by *miR156* [50]. Indeed, the importance of *miR156* in the juvenile-to-adult phase transition has been recently demonstrated in some trees [61]. In *Arabidopsis*, *SPLs* provide a gene family of 16 members, 10 of them regulated by *miR156* [62]. The citrus *SPL*-like seems to be a close relative of the small *SPL* genes, *SPL3/4/5*, based on three criteria: first, like *SPL3/4/5*, its *miR156*-binding site is located within the 3′UTR and not within the coding region as in other *SPL* family members (Shalom L, Shlizerman L, Blumwald E, Tumimbang E, Sadka A, in preparation); second, the putative SBP domain of citrus *SPL*-like shows highest homology to those of the *Arabidopsis SPL3/4/5*; third, similar to *SPL3/4/5*, its expected product is relatively small (130 amino acids). This gene's overall expression pattern in the buds suggests that it is negatively regulated by fruit load, and therefore might play a role in flowering induction following an OFF year. Functional analysis of the citrus gene in *Arabidopsis* showed that its overexpression induces early flowering, and that it possesses an active *miR156*-binding site (Shalom L, Shlizerman L, Blumwald E, Tumimbang E, Sadka A, in preparation). In *Arabidopsis*, SPLs act in both the leaf and the apical meristem to promote flowering in a complex manner involving several pathways [50]. In one of them, operated in the apical meristem, SPL3 and SPL9 bind to the promoter regions of flower meristem identity genes, inducing their expression. In this way, SPL promotes the expression of *FUL* and *LFY*, SPL9 promotes the expression of *SOC1* and *AGL42*, and both SPL3 and SPL9 promote the expression of *AP1*, in concert with the FT/FD complex [63]. The overall expression pattern of *SPL*-like in OFF buds compared to ON buds suggests that the gene responds to fruit load; the highest difference in mRNA levels, about 4-fold, was detected in May. This provides further support for the hypothesis that ‘AB signal’ is generated early in the season, at least 6 months prior to the flowering induction period. However, considering the action of SPLs in flowering induction, the overall reduction in this gene's mRNA levels is somewhat surprising. In fact, its mRNA levels are minimal during the flowering induction period, from November to January, when genes downstream of *SPL*—*LFY*, *SOC1*, *FT2* and *AP1*—are induced. It might be that SPL itself is regulated at the post-transcriptional level. Although less likely, it might be that in contrast to *Arabidopsis*, SPL does not act directly on the expression of flowering control genes, but generates a signal which acts during the flowering induction period. In any case, as already discussed, these results further emphasize the complexity in interpreting expression patterns. Further complexity stems from the pattern of expression of *miR156*: *miR256* was induced from September to January, in accordance with the reduction in *SPL* expression. However, its levels were slightly reduced from May to September, when *SPL* mRNA levels were also reduced. Moreover, no difference in its levels was detected between ON and OFF buds. These results suggest that *SPL* might be subjected to other modes of regulation, an option that is currently being investigated in our laboratory.

### Differentially expressed pathways in ON and OFF trees

The clustering analysis demonstrated that the impact of the three tested conditions, time, tissue type and AB state, on the level of similarity between the expression profiles follows the order: AB state>tissue type>time, i.e., ON and OFF organs showed more similar patterns than under the effect of time or tissue type (Figure 4A). This means that developmental changes over time within the same organ resulted in greater changes in gene expression than ON and OFF states of the organ at the same time point. However, it should also be noted that buds and LS showed more similar patterns at the same time point than buds or LS at different times. A similar gene expression pattern suggests that buds and LS share common functions, which is not surprising considering the fact that the bud contains leaf primordia.

Among the three analyzed time points, the largest number of DEPs between ON and OFF trees was detected in May, while in September and between November and January, their number was relatively low. Moreover, in May, the number of DEPs was much higher in buds than in LS. Taken together with the finding that the maximal difference in bud width develops between May and July, these results suggest that AB signal, if present, is generated much earlier than the flowering induction period, and causes the above changes. Alternatively, changes in gene expression in May might reflect changes in resource allocation: when fruit is present (ON trees), buds are deprived of photoassimilates, which directly reduces their size in comparison to the case in OFF trees.

### Biological processes affected by fruit load in May buds

As already noted, the highest number of DEPs was evident during May in the buds. Obviously, we cannot cover all of the metabolic processes which are induced at this time point, and we therefore briefly discuss three of these processes: two of them, flavonoid biosynthesis and photosynthesis, are induced in OFF buds, and one, trehalose metabolism, is induced in ON buds. The expression of genes belonging to two of these processes, flavonoid biosynthesis and trehalose metabolism, were also validated by nCounter technology.

#### Trehalose metabolism

ON buds showed increased expression of the two genes of trehalose metabolism, *TPS* and *TPP*. Trehalose is a disaccharide, which serves as an alternative sugar to sucrose in a variety of bacterial and fungal species [64]. In resurrection plants, where it serves as an osmoprotectant, trehalose is present at high levels, but usually in higher plants it is below detection levels. Changes in the trehalose biosynthetic genes and/or enzymes, and not necessarily trehalose levels themselves, were postulated to play a signaling or regulatory role in stress-response pathways [65]. Moreover, *Arabidopsis* plants mutated in *TPS* show arrested-growth phenotypes, remaining in the vegetative growth phases, suggesting that the gene is required for proper embryo development [66]. These results demonstrate the importance of the trehalose biosynthetic pathway for normal vegetative growth and transition to the flowering phase. An increase in the expression of TPS and TPP along with unchanged expression in trehalase, which catabolizes trehalose, suggests that trehalose level and/or pathway is induced in ON buds in May. These results can be explained in two ways. First, as a result of the high investment in developing fruits, ON trees are commonly under stress [7], which might directly affect the bud. Increased production of trehalose might play a role in mitigating the effects of these stresses. Second, the significant differences in *TPS* and *TPP* expression in buds, but not in LS, in May suggest a possible role for trehalose and/or its biosynthetic pathway in citrus flowering induction and in the regulation of AB itself, via an unknown mechanism.

#### Photosynthesis

Genes belonging to the three components of photosynthesis—light reactions, Calvin cycle and photorespiration—are induced in OFF buds (Figure S3, Table S6). In addition, SEA of expression showed that processes involved in the detection of light stimulus (including red/far red light) and phototransduction are also induced in OFF buds (Table 1). Bud morphology does not allow efficient photosynthesis and like the fruit, it provides a sink organ for photoassimilates. Moreover, while in ON trees, the bud competes with the developing fruit for resources, no such competition occurs in OFF trees, loaded with photoassimilates and storage molecules. In fact, according to nutritional theory, photoassimilate availability might well play a regulatory role in flowering induction. However, one could question the reason for inducing the photosynthetic machinery within the bud in an OFF year. We hypothesize that this induction provides a signal for the nutritional status of the bud. In other words, the bud signals its surrounding source leaves that it is loaded with photoassimilates, so the translocation rate is reduced. Although at this stage we cannot provide direct evidence for this hypothesis, it has been suggested that specific tissues within tomato fruit signal their sink strength by altering their photosynthetic machinery; indeed, different tissues possess different photoassimilate-translocation rates.

#### Flavonoid biosynthesis

Genes of a few pathways of secondary metabolism were induced in OFF buds, including flavonoids, phenylpropanoids, alkaloids and lignin (Table 1, Figure 5, Figure S6). Induction of five flavonoid biosynthesis genes was validated by nCounter technology, and metabolomic analysis confirmed that the pathway might indeed be induced in OFF buds in May (Figure 9, Figure S6). The induction of specific flavonoids was relatively marginal; however, the identification was limited by the standards used, and other flavonoids might also be induced. In any case, it seems that not only the central pathway was induced, but also the side reactions. Flavonoids are secondary metabolites that influence a variety of characteristics, such as aroma and flavor pigmentation, as well as protection against UV radiation [67]. Their synthesis has been hypothesized to occur under conditions of excess photoassimilates, particularly sucrose [68]; sucrose feeding of *Arabidopsis* plants has been shown to result in increased expression of flavonoid biosynthetic genes, especially those encoding anthocyanin [69]. In light of these findings, it is suggested that flavonoids in the bud serve as “sink” molecules for excess photoassimilates and other carbon molecules accumulating in the tree in OFF years.

In summary, results of this work show that a relatively long time before the flowering induction period, fruit load affects many regulatory and metabolic processes in the bud. Obviously, it should be considered that this and other conclusions of the work are based on a single cropping year. Although the expression of some of the flowering control genes was partially investigated during another year, with similar results (data not shown), year to year environmental and other external variations might affect the results, and therefore the conclusions. It should also be mentioned that the nature of the AB signal, and whether it is produced that early, remain open questions. Even if produced in May, or earlier, the signal must be reversible, as fruit thinning or complete removal from ON trees reverses the AB state. Ongoing studies in our laboratory include analyses of buds following fruit removal in September, when the number of differentially expressed genes between ON and OFF buds was very low. These analyses are expected to clarify which of the processes induced in ON and OFF buds are directly affected by fruit load. We are also investigating the possibility of *SPL*-like playing a role in AB signaling. In light of suggestions in the literature, the trehalose metabolism is involved in vegetative and reproductive growth, and the possible involvement of this metabolism in AB control warrants further study.

## Supporting Information

Figure S1 **The annual cycle in citrus.** Stage I and Stage II of fruit development are as described previously [41].
(TIF)

Figure S2 **ON and OFF shoots in citrus.** Schematic description of OFF-year fruitless shoot and ON-year fruit bearing shoot. Buds are represented as brown triangle. Bud collection from OFF shoot was performed as described under Material and Methods. All buds of ON shoot were collected for the analyses.
(TIF)

Figure S3 **Induction of photosynthesis in OFF buds.** Differentially expressed probes were analyzed by MapMan. Blue squares represent genes induced in ON buds and red squares represent genes induced in OFF buds. A description of the specific genes and their fold change is provided in Table S6.
(TIF)

Figure S4 **Expression of trehalose metabolism genes in ON and OFF buds.** mRNA levels (RE) of trehalose phosphate phosphatase (TPP) and trehalose phosphate synthase (TPS) were measured in ON and OFF buds during the indicated months.
(TIF)

Figure S5 **Expression of flavonoid biosynthetic pathway genes in ON and OFF buds.** mRNA levels of phenylalanine ammonia-lyase (*PAL*), chalcone synthase (*CHS*), cinnamate 4-hydroxylase (*C4H*), 4-coumarate:coenzyme A ligase (*4CL*), chalcone isomerase (*CHI*), flavanone 3-hydroxylase (*F3H*), dihydroflavonol 4-reductase (*DFR*), isoflavone reductase (*IFR*), flavonol synthase (*FLS*), Anthocyanidin synthase (*AS*) and UDP-glucose:flavonoid-3-O-glucosyltransferase (*UF3GT*) were measured in ON and OFF buds during the indicated months.
(TIF)

Figure S6 **Induction of flavonoid pathway in OFF buds.** A scheme showing the biosynthetic pathway of flavonoids. Genes induced in OFF buds in the microarray or in the real-time PCR are marked with squares. Standards for specific flavonoid groups are also marked.
(TIF)

Table S1 **Primers list.**
(XLSX)

Table S2 **List of genes (** http://www.phytozome.net/ **) used for nCounter analysis.**
(XLSX)

Table S3 **Microarrays hybridization results by log signal.**
(XLSX)

Table S4 **GO annotations for differentially expressed probes in May buds.**
(XLSX)

Table S5 **Probe list of** **Fig. 5** **.**
(XLSX)

Table S6 **Probe list of Fig. S3.**
(XLSX)
