# Supplementary figures and images for: Alternate Bearing in Citrus: Changes in the Expression of Flowering Control Genes and in Global Gene Expression in ON- versus OFF-Crop Trees
Source: PLoS One. 2012 Oct 11;7(10):e46930. doi: 10.1371/journal.pone.0046930 (PMC3469648; doi:10.1371/journal.pone.0046930)

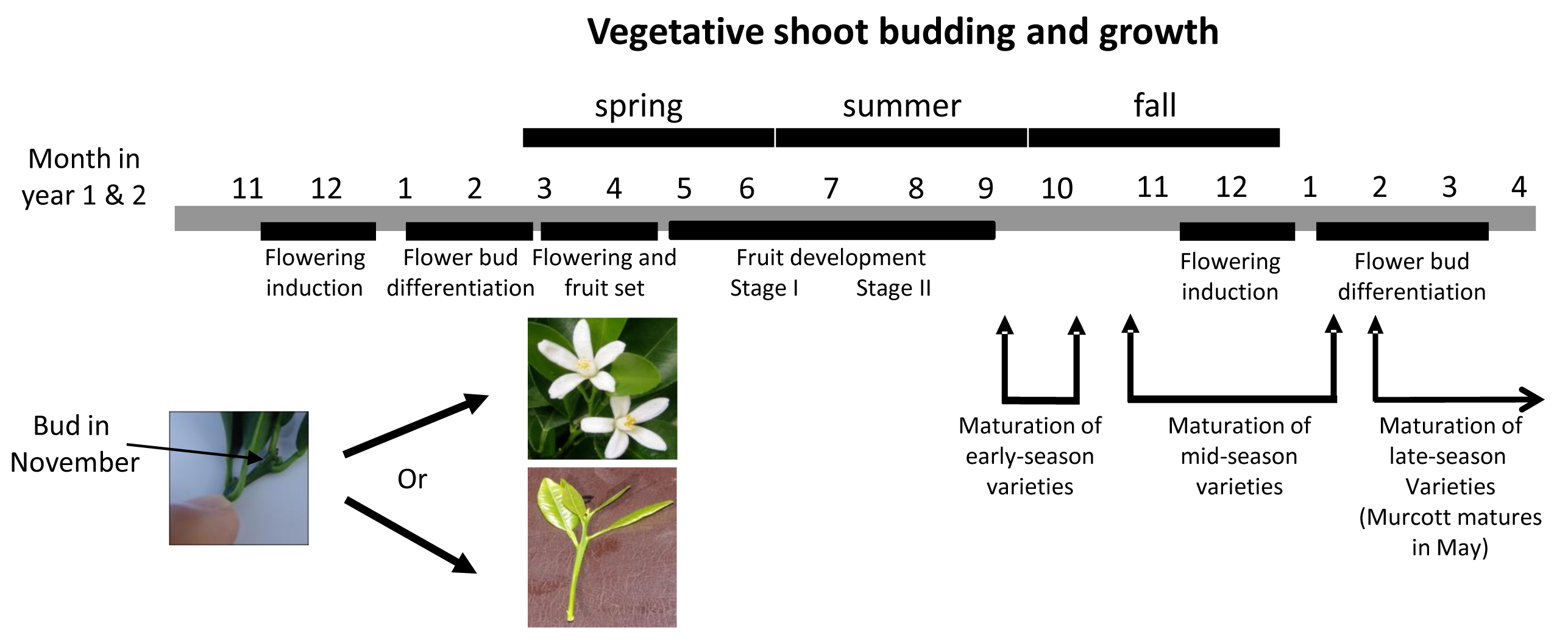

Supplement: Figure S1 — The annual cycle in citrus. Stage I and Stage II of fruit development are as described previously [41]. (TIF) [file pone.0046930.s001.tif]

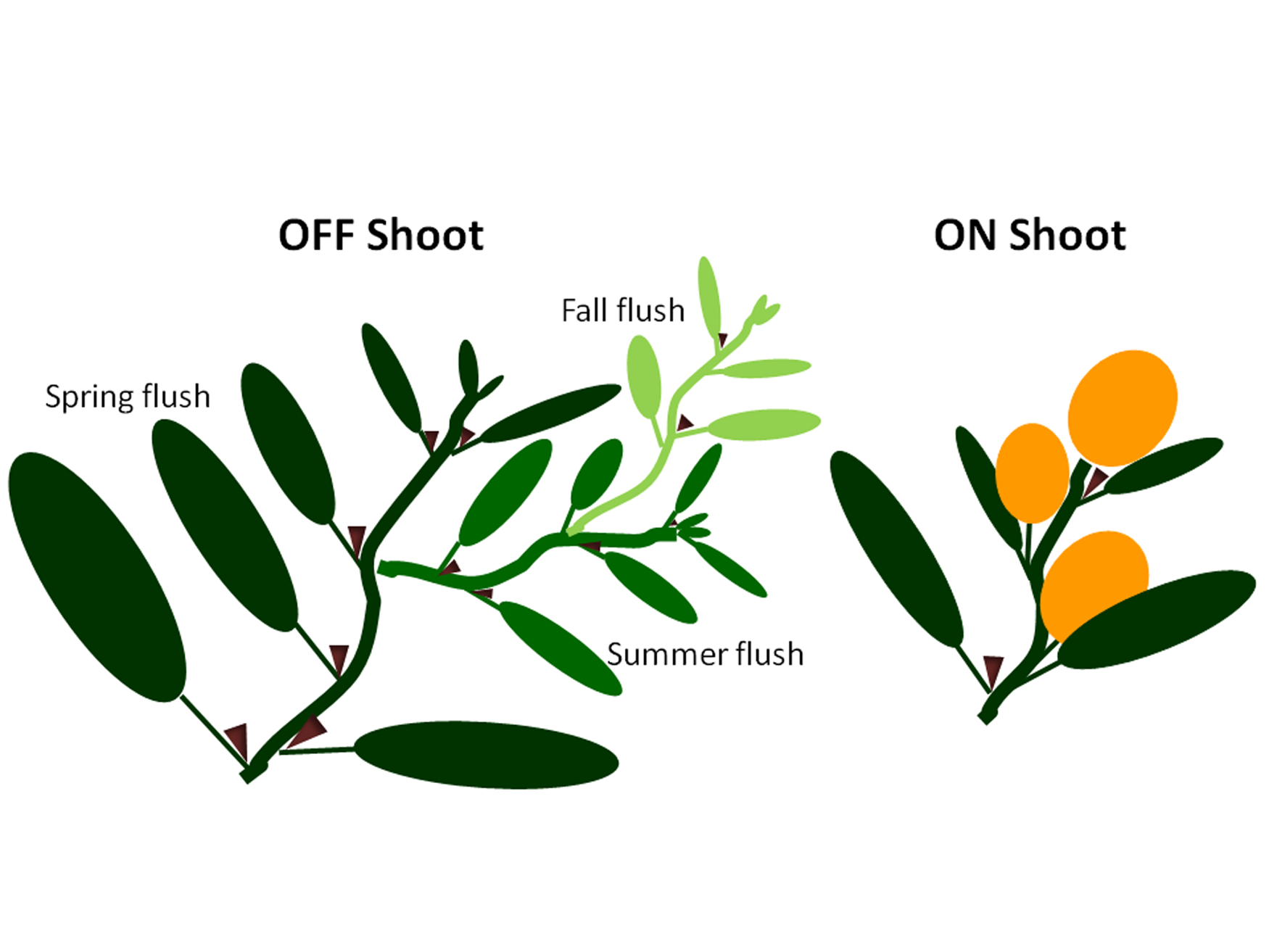

Supplement: Figure S2 — ON and OFF shoots in citrus. Schematic description of OFF-year fruitless shoot and ON-year fruit bearing shoot. Buds are represented as brown triangle. Bud collection from OFF shoot was performed as described under Material and Methods. All buds of ON shoot were collected for the analyses. (TIF) [file pone.0046930.s002.tif]

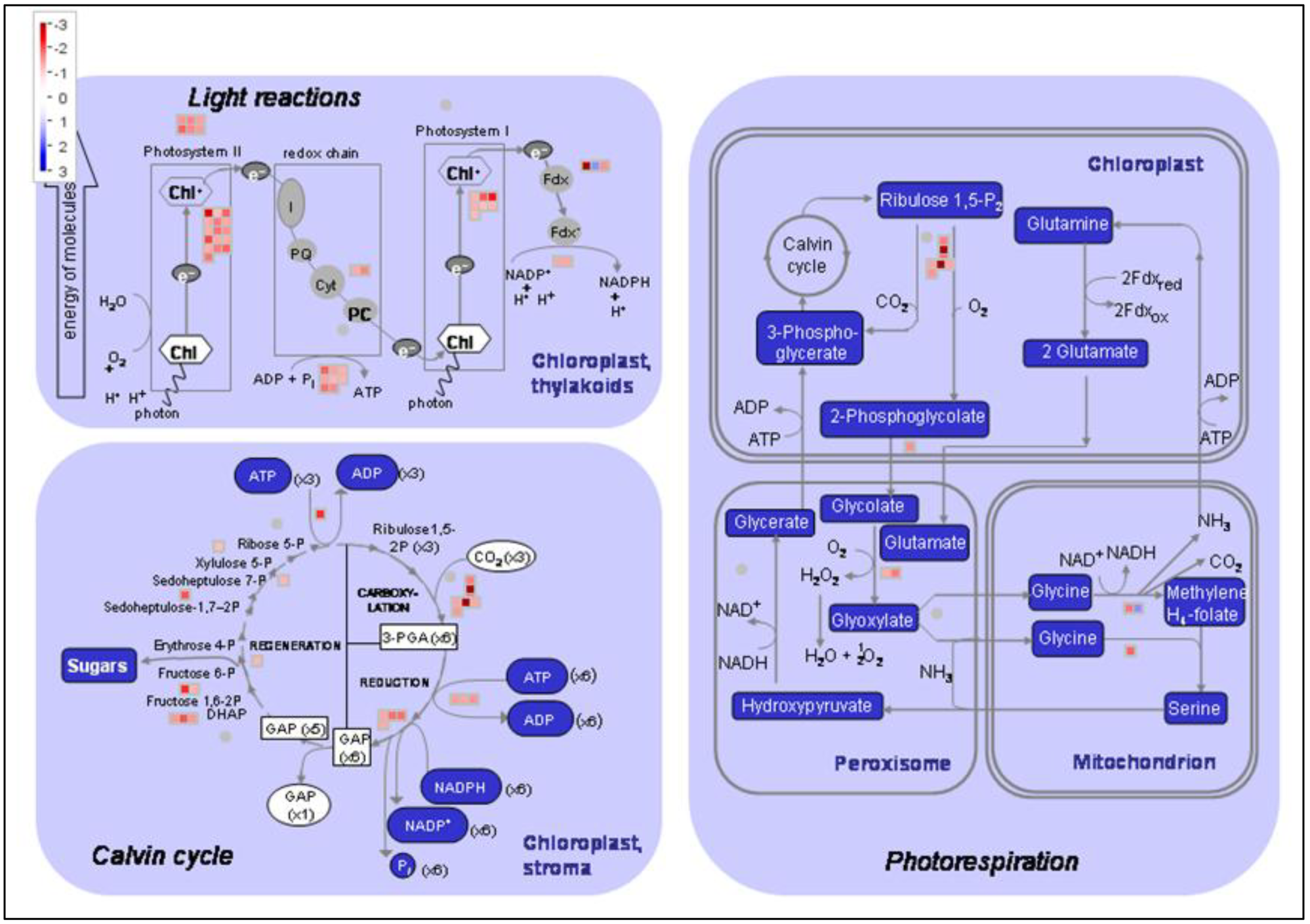

Supplement: Figure S3 — Induction of photosynthesis in OFF buds. Differentially expressed probes were analyzed by MapMan. Blue squares represent genes induced in ON buds and red squares represent genes induced in OFF buds. A description of the specific genes and their fold change is provided in Table S6. (TIF) [file pone.0046930.s003.tif]

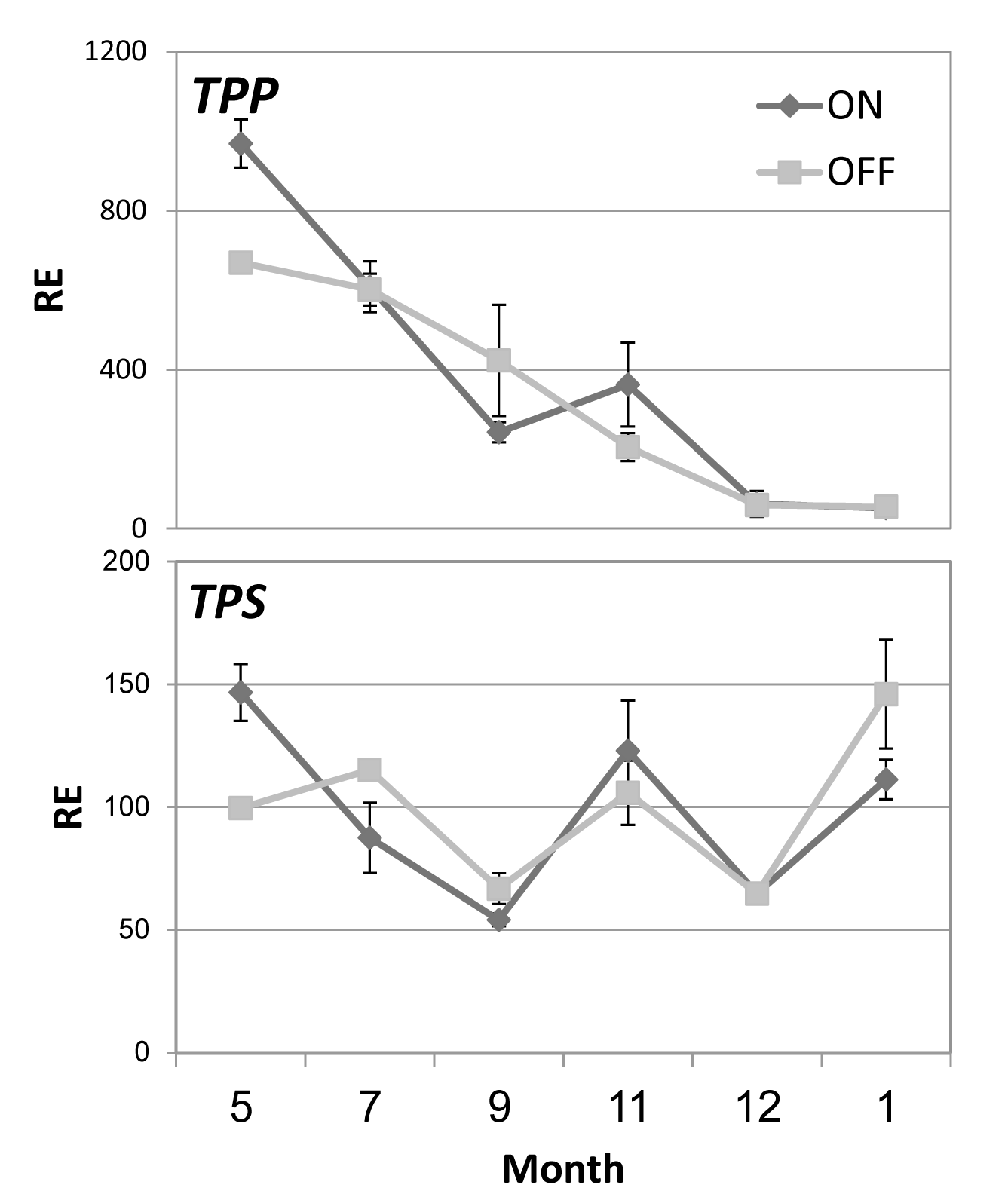

Supplement: Figure S4 — Expression of trehalose metabolism genes in ON and OFF buds. mRNA levels (RE) of trehalose phosphate phosphatase (TPP) and trehalose phosphate synthase (TPS) were measured in ON and OFF buds during the indicated months. (TIF) [file pone.0046930.s004.tif]

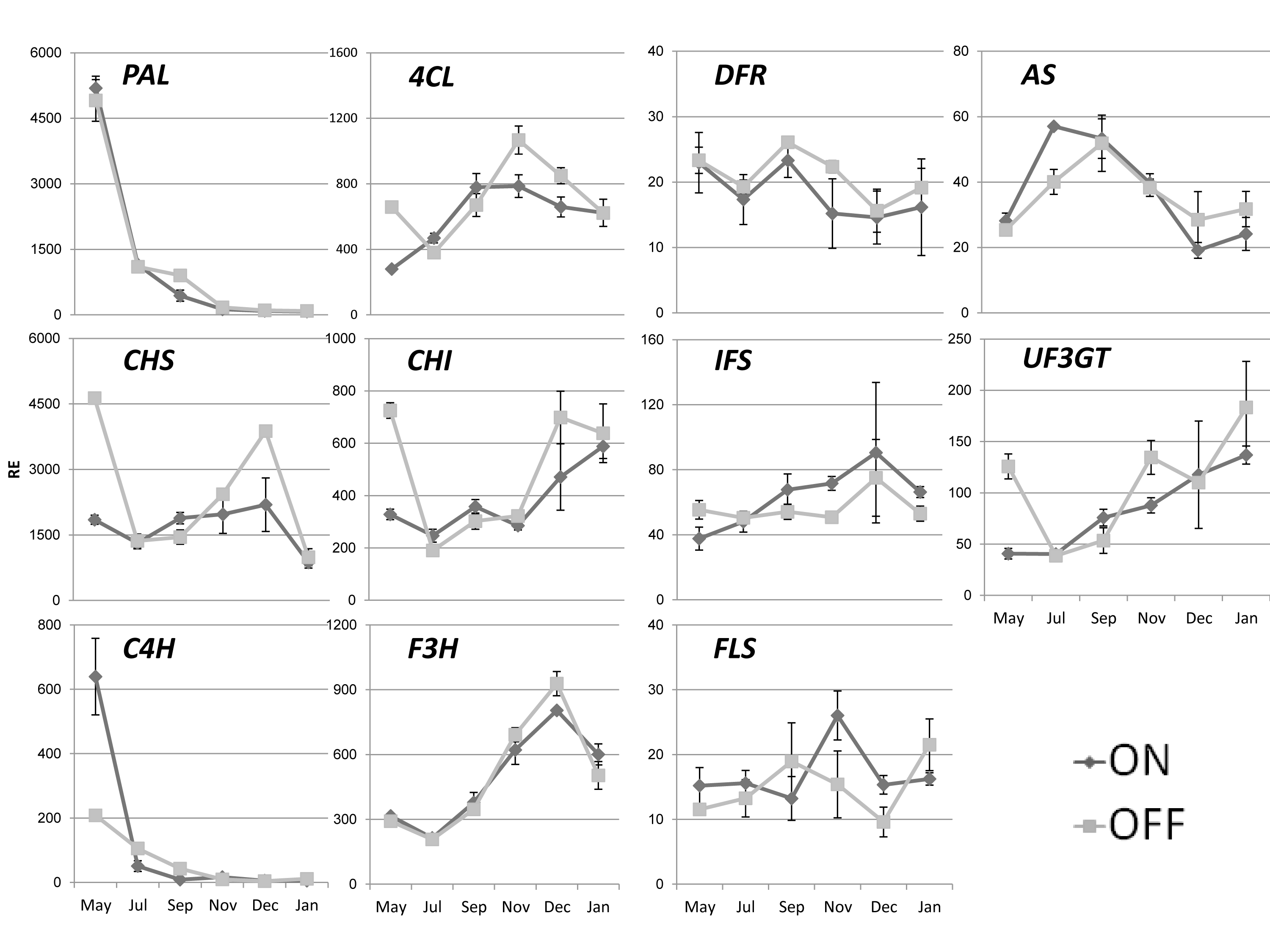

Supplement: Figure S5 — Expression of flavonoid biosynthetic pathway genes in ON and OFF buds. mRNA levels of phenylalanine ammonia-lyase (PAL), chalcone synthase (CHS), cinnamate 4-hydroxylase (C4H), 4-coumarate:coenzyme A ligase (4CL), chalcone isomerase (CHI), flavanone 3-hydroxylase (F3H), dihydroflavonol 4-reductase (DFR), isoflavone reductase (IFR), flavonol synthase (FLS), Anthocyanidin synthase (AS) and UDP-glucose:flavonoid-3-O-glucosyltransferase (UF3GT) were measured in ON and OFF buds during the indicated months. (TIF) [file pone.0046930.s005.tif]

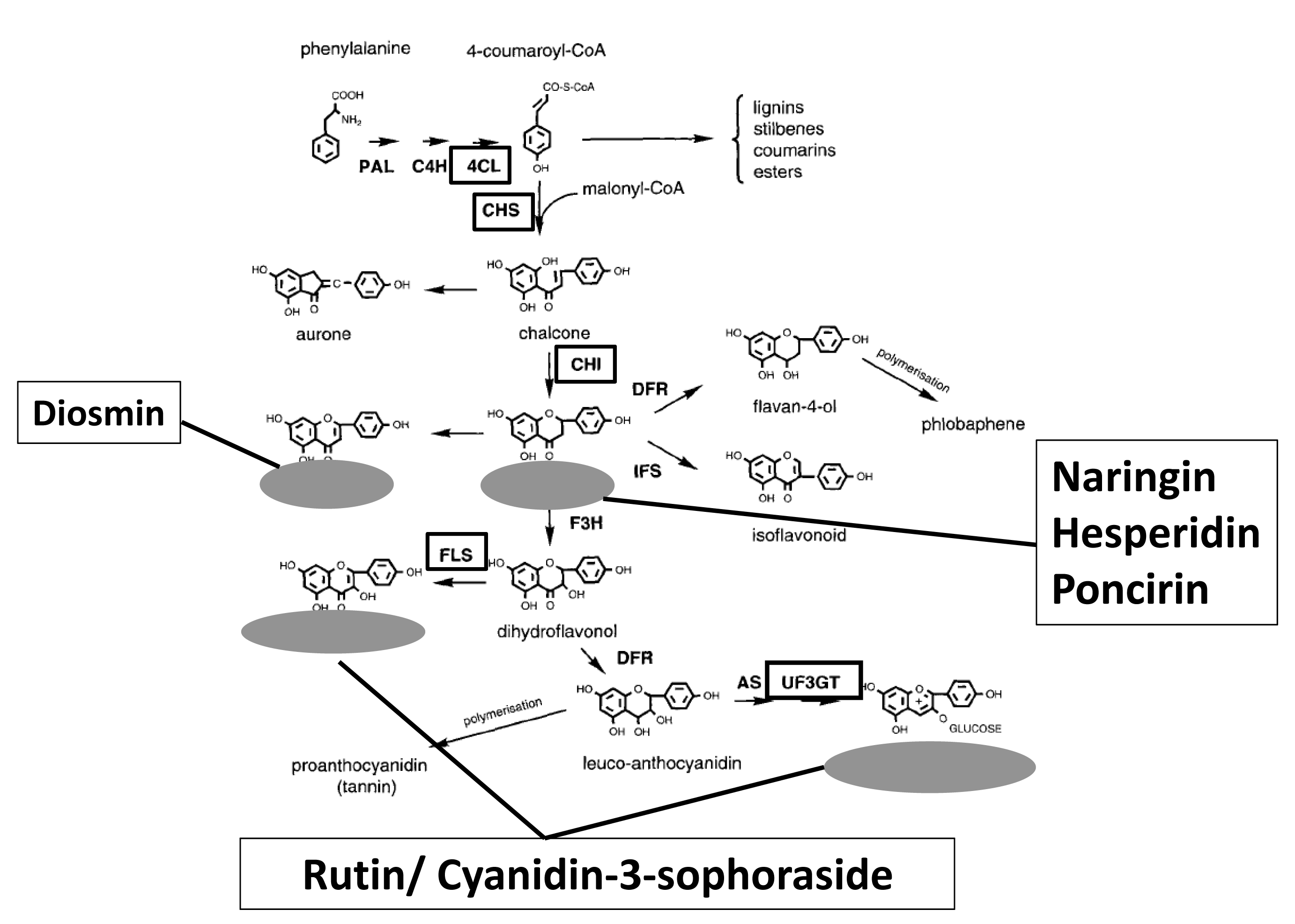

Supplement: Figure S6 — Induction of flavonoid pathway in OFF buds. A scheme showing the biosynthetic pathway of flavonoids. Genes induced in OFF buds in the microarray or in the real-time PCR are marked with squares. Standards for specific flavonoid groups are also marked. (TIF) [file pone.0046930.s006.tif]
